# Genome-wide association study reveals the genetic architecture of root hair length in maize

**DOI:** 10.1186/s12864-021-07961-z

**Published:** 2021-09-14

**Authors:** Lin Liu, Lu-Guang Jiang, Jin-Hong Luo, Ai-Ai Xia, Li-Qun Chen, Yan He

**Affiliations:** 1 State Key Laboratory of Plant Physiology and Biochemistry, College of Biological Sciences, China Agricultural University, Beijing, 100193 China; 2 MOE Key Laboratory of Crop Heterosis and Utilization, National Maize Improvement Center of China, China Agricultural University, Beijing, 100193 China

**Keywords:** Maize, Root hair, GWAS, SNPs

## Abstract

**Background:**

Root hair, a special type of tubular-shaped cell, outgrows from root epidermal cell and plays important roles in the acquisition of nutrients and water, as well as interactions with biotic and abiotic stress. Although many genes involved in root hair development have been identified, genetic basis of natural variation in root hair growth has never been explored.

**Results:**

Here, we utilized a maize association panel including 281 inbred lines with tropical, subtropical, and temperate origins to decipher the phenotypic diversity and genetic basis of root hair length. We demonstrated significant associations of root hair length with many metabolic pathways and other agronomic traits. Combining root hair phenotypes with 1.25 million single nucleotide polymorphisms (SNPs) via genome-wide association study (GWAS) revealed several candidate genes implicated in cellular signaling, polar growth, disease resistance and various metabolic pathways.

**Conclusions:**

These results illustrate the genetic basis of root hair length in maize, offering a list of candidate genes predictably contributing to root hair growth, which are invaluable resource for the future functional investigation.

**Supplementary Information:**

The online version contains supplementary material available at 10.1186/s12864-021-07961-z.

## Background

Root hairs are special tubular-shaped outgrowth from root epidermal cells, which vastly enlarge the root surface area and assist in water and nutrient absorption such as NO^3−^, Cl^−^, Ca^2+^, K^+^, Zn^2+^, and Mn^2+^, as well as the interactions with biotic and abiotic stresses [1, 2]. On the other hand, the length, density and morphology of root hair are influenced by various endogenous and environmental factors, including phytohormones and mineral nutrients, especially under phosphate (Pi) limiting condition [3–7]. The development of root hair can be separated into three basic stages: specification of the epidermal cell fate, initiation of the root hair outgrowth, and elongation of the hair via tip growth [2, 8].

As a unique single cell type in plant biology, the development, physiology, and cell biology of root hair have been intensively studied in *Arabidopsis* [2, 6, 9–11]. In contrast, only a few of genes functional in root hair development have been known in monocot crop species [12]. In rice, some RSL class I and RSL Class II genes have been reported to positively regulate the development of root hairs, suggesting that the mechanism of RSL-regulated root hair development is at least partly conserved among grasses and eudicots [13, 14]. In addition, several genes involved in the elongation of root hairs also have been identified in rice, including *OsEXPA8* [15], *OsEXPA17* [16], *OsCSLD1* [17], *OsFH1* [18], *OsSNDP1* [19] and *OsPHR* [20]. So far, a total of six genes involved in root hair development have been identified in maize. *Roothairless 1* (*rth1*), *rth3*, *rth5* and *rth6* mutants are deficient in root hair formation in all root types, and exhibit defect in different stages of root hair development [21–24]. The *ZmLRL5* gene, encoding a basic helix–loop–helix (bHLH) transcription factor, was demonstrated to play a positive role in orchestrating the translational process by directly regulating the expression of translational processes/ribosomal genes during maize root hair growth. The loss-of-function of *ZmLRL5* resulted in a dramatic reduction in the elongation of root hair [25]. Recently, *ZmTIP1*, encoding a functional S-acyltransferase, was identified to participate in drought tolerance by regulating root hair growth [26]. Although these reports have made great contribution to understand molecular regulation of root hair development in maize, it remains unclear how the root hair growth is controlled in a natural population.

In recent years, genome-wide association studies (GWAS), which is based on linkage disequilibrium (LD) in a panel, has offered high mapping resolution and could effectively benefit the exploration of the genetic basis associated with complex quantitative traits [27–30]. In maize, LD decay is rapid due to its extensive genetic diversity. Therefore, maize is recognized as an ideal model plant for conducting association studies [31–34]. To date, GWAS has successfully used to identify numerous candidate loci/genes controlling sever-al morphological or metabolic traits in maize, such as shoot apical meristem size, husk trait, plant height, kernel weight, drought tolerance, grain drying rate and grain moisture [35–41]. In this study, we used a maize association panel including 281 inbred lines with tropical, subtropical, and temperate backgrounds to interpret the phenotypic diversity and the genetic basis of root hair development. Several candidate genes putatively involved in root hair development were identified, providing a useful resource for further functional studies to elucidate molecular pathways involved in maize root hair growth and development.

## Results

### Phenotypic variation among root hair length

**Fig. 1:**
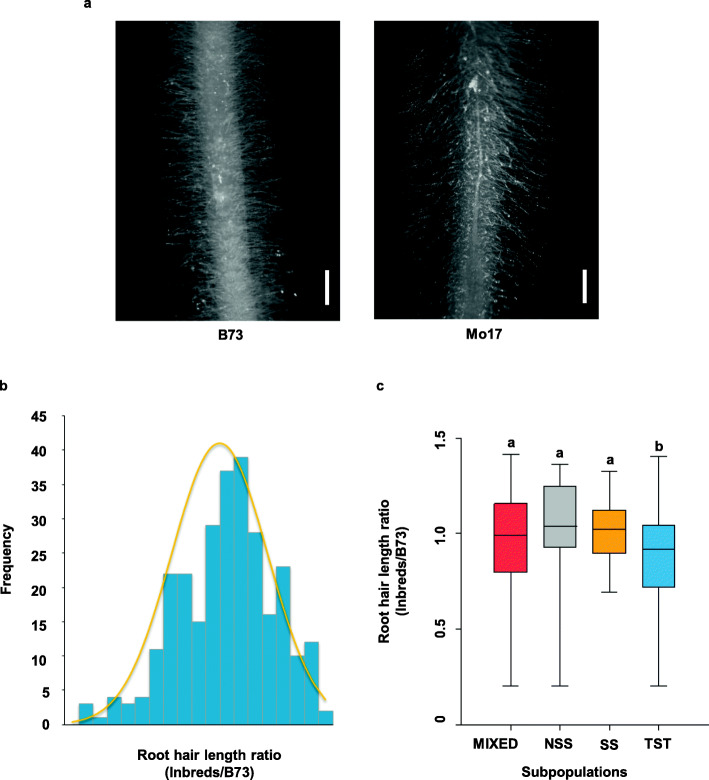
Phenotypic variation of root hair length. **a** Diagram of B73 (left) and Mo17 (right) root hair length. Bars = 1 mm. **b** Frequency distributions of root hair length. Classes of trait values are shown on X axis and counts of inbred lines with the phenotypic values for these bins are shown on Y axis. **c** Boxplot of root hair length distribution in different subpopulations. Kruskal-Wallis test was applied to examine the difference of traits among subpopulations. Different letters indicate significance levels at *P* ≤ 0.05. No. of inbred lines included in each subpopulation are 117, 30, 22 and 112 for MIXED, NSS, SS and TST, respectively

The association population in this study consists of a global collection of 281 diverse maize inbred lines [42, 43]. The root hair length of primary roots was measured from 3-day-old plants (Table S1). The noticeable variation in root hair length was represented by B73 and Mo17, two inbred lines commonly used in maize biology (Fig. 1a). The measured root hair trait followed a normal distribution with a slight right skew (Fig. 1b). The trait maximum, minimum, mean, standard deviation and coefficient of variation were listed in Table 1. The ratio of root hair length of inbred lines to B73 control ranged from 0.2 to 1.42 with the mean of 0.95, indicating that the root hair length exhibits broad variations in the association population.

**Table 1:** Phenotypic statistics of root hair trait for the 281 inbred lines

Trait^a^	Minimum	Maximum	Mean	SD^b^	CV^c^
Ratio	0.20	1.42	0.95	0.24	0.2526

^a^ The ratio of root hair length of inbred lines to B73 control

^b^ Standard deviation

^c^ Coeffcient of variation

All 281 lines used in this study include three subpopulations and one mixed group, which are referred to TST, SS, NSS, and MIXED [42]. TST subpopulation is of tropical or subtropical origin, consisting of 112 lines. SS and NSS subpopulations are of temperate origin, consisting of 22 and 30 lines, respectively. MIXED subpopulation is inbred lines which were not accurately assigned into the above three subpopulations based on the phylogenic analysis [42, 43]. To investigate the effect of population structure on root hair phenotypes, the root hair length was compared between different subpopulations. Compared with SS, NSS and MIXED subpopulations, the mean value of TST subpopulation was significantly less, suggesting that maize inbred lines from tropical/subtropical origin tend to have shorter root hairs (Fig. 1c).

### Associations of root hair phenotype with agronomic traits and metabolic pathways

As root hairs play a crucial role in the plant acquisition of nutrients and water, we postulated that root hair morphology is likely coordinated with other agronomic traits and amino acid metabolism. To verify our hypothesis, the Pearson-correlations were calculated after comparing root hair length with 17 agronomic traits and 18 amino acid contents in maize kernel, which were previously measured in the same association panel [44, 45]. The 17 agronomic traits include seven morphological traits, i.e. plant height (PH), ear height (EH), ear leaf width (ELW), ear leaf length (ELL), tassel maximum axis length (TMAL), tassel branch number (TBN), leaf number above ear (LNAE); seven yield-related traits, i.e. ear length (EL), ear diameter (ED), cob diameter (CD), kernel number per row (KNPR), cob grain weight (GW), cob weight (CW), kernel width (KW); three flowering-related traits, i.e. days to anthesis (DTA), days to silking (DTS) and days to heading (DTH) [44]. The amino acids measured in dry maize kernel include Ala, Arg, Asx, Glx, Gly, Lle, Leu, Lys, Met, Pro, Phe, Val, Tyr, His, Cys, Thr and Ser, and the total concentration of amino acids [45].

**Fig. 2:**
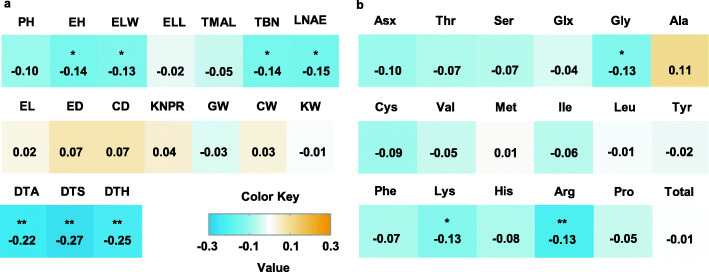
Correlation coefficients of root hair length with other agronomic and amino acidmetabolic traits. **a** Correlation coefficients between root hair length with 17 agronomic traits. **b** Correlation coefficients between root hair length with 18 metabolic traits. *Significant at *P* ≤ 0.05; **significant at *P* ≤ 0.01. The correlation level is color-coded according to the color key plotted in the middle

Of the 17 agronomic traits examined, 7 were correlated with root hair length (*P* ≤ 0.05), which were EH, ELW, TBN, LNAE, and all three flowering-related traits (Fig. 2a). Meanwhile, of the 18 amino acids, 3 were correlated with root hair length (*P* ≤ 0.05), which were Gly, Lys and Arg (Fig. 2b). The Pathway Association Study Tool (PAST) was further performed to elucidate the biochemical pathways likely contributing to root hair elongation [46–48]. Under the statistical threshold *P* < 0.01, a total of 8 and 1 metabolic pathways exhibited significant associations with increased or decreased root hair length, respectively (Table S2 and S3). Overall, the close correlations of root hair length with other agronomic or metabolic traits above-mentioned suggest that the root hair occurrence is correlated to other aspects of plant development and growth.

### GWAS on maize root hair length

**Fig. 3:**
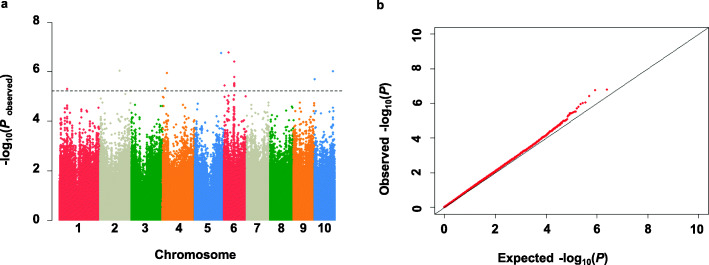
GWAS showing significant *P*-values associated with root hair length based on the mixed linear model (MLM). **a** Manhattan plots of MLM for root hair length. The horizontal dashed line represents the significance threshold -log10(*P*) = 5.2. **b** QQ plot of MLM showing the ratio of the observed and the expected *P*-values for root hair length. The solid diagonal lines represent agreement between observed and expected probability distributions assuming null SNP–trait association

Using 1,253,814 SNPs covering the whole maize genome with a minor allele frequency (MAF) ≥ 0.05, we performed GWAS to explore the genetic loci underlying the root hair length. Under the mixed linear model (MLM) [49, 50], which accounts for false positives arising from the population structure (Q matrix) and kinship relationship (K matrix) of the natural variation in the population, a stringent threshold of –log_10_*P* ≥ 5.2 was designated as the threshold for calling significantly associated SNPs. The Manhattan plots for the SNPs associated with root hair length were shown in Fig. 3. In total, 18 significant SNPs were detected, which are located on chromosomes 1, 2, 4, 5, 6, and 10, explaining the phenotypic variations ranged from 6.3 to 10.1%. Moreover, GWAS was also performed using the general linear model (GLM). Under the stringent threshold of –log_10_*P* ≥ 6.5, a total of 14 SNPs were identified, 12 out of which overlapped with MLM-derived SNPs (Fig. S1). As there were 8 MLM-derived SNPs on chromosome 6 present within the same LD region (*r*^*2*^ ≥ 0.2), and the leading SNP_ 75630277 was selected as the representative of this locus (Table 2), yielding a total of 11 SNPs used for the further analysis.

**Table 2:** SNPs, chromosomal position and candidate genes significantly associated with root hair trait identified by GWAS using MLM

Chr	Position (bp)	Allele	R^2^ (%)^a^	MAF^b^	*P*-value	Gene	Gene interval (bp)	Annotation
1	55388394	T/C	8.0	0.08	5.16E-06	GRMZM2G100288	55388285–55391652	Receptor-like protein kinase (FERONIA)
2	148870290	C/T	10.1	0.09	9.39E-07	GRMZM2G147446	148863858–148867640	UDP-rhamnose/UDP-galactose transporter 5
2	229021546	G/A	9.3	0.07	5.81E-06	GRMZM2G078013	229020754–229026704	NBS-LRR disease resistance protein
4	20054633	C/G	6.3	0.06	4.79E-06	GRMZM2G064644	20054312–20054951	ROP-interactive CRIB motif-containing protein (RIC2)
4	34337601	C/T	9.3	0.07	1.15E-06	GRMZM2G000471	34335083–34336530	Aquaporin NIP5–1
5	198197360	A/C	9.8	0.05	1.80E-07	GRMZM2G044851	198193875–198198340	Nitrate transporter 1.5
6	5509266	A/G	7.4	0.05	3.65E-06	AC193598.3_FG002	5503430–5507323	NBS-LRR disease resistance protein
6	34221678	T/A	9.0	0.08	1.70E-07	GRMZM2G403003	34217717–34222233	TON1 recruiting motif 19 (TRM19)
6	75630277	A/G	9.7	0.06	3.97E-07	GRMZM5G825276	75630353–75630906	GDSL-like lipase
10	2049694	C/A	9.2	0.16	2.00E-06	GRMZM2G180244	2049440–2055121	NBS-LRR disease resistance protein
10	138881367	T/G	9.9	0.29	9.85E-07	GRMZM2G091579	138880717–138881634	Uncharacterized protein

^a^ Percentage of phenotypic variation explained by the additive effect of the single significant SNP

^b^ Minor allele of frequency

### Genes co-localized with significant SNPs

**Fig. 4:**
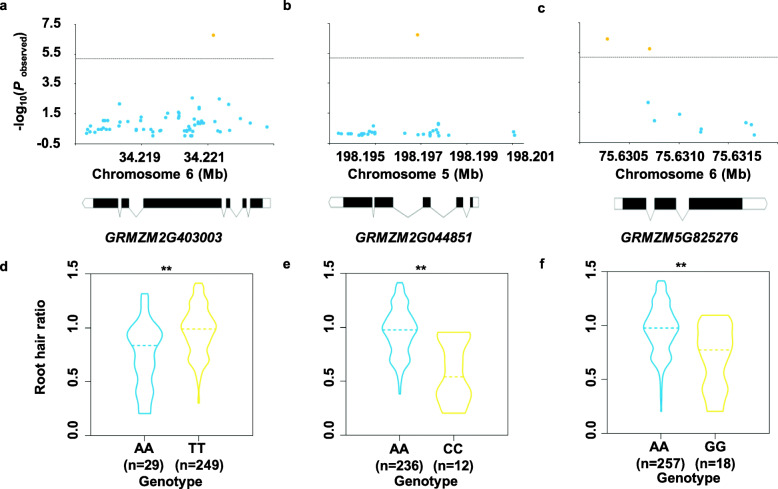
The allele effects of significant SNPs located around representative genes for root hair length. **a**–**c** Regional plots showing association mapping results for SNPs located around *GRMZM2G403003* (**a**), *GRMZM2G044851* (**b**), *GRMZM5G0825276 *(**c**). **d**–**f** Allele effects of the most significant SNPs for root hair traits. *Significant at *P* ≤ 0.05; **significant at *P* ≤ 0.01. (**d**) *GRMZM2G403003*, (**e**) *GRMZM2G044851*, (**f**) *GRMZM2G825276*. Each dot represents an SNP. The horizontal dashed black line represents the significant threshold –log10(*P*) = 5.2

Protein-coding genes harboring or nearest to the significant SNPs were nominated as the candidates associated with root hair (Table 2). The allelic effect of haplotype coordinated with significant SNPs on root hair phenotypes was assessed (Fig. 4 and Figure S2). The most significant SNP (chr6.S_ 34221678, *P*-value = 1.70E-07, *R*^2^ = 9.0%) locates in the fourth exon of GRMZM2G403003 (Fig. 4a), encoding a TON1 recruiting motif (TRM) protein. The average root hair length for A allele was substantially shorter than T allele (*P* ≤ 0.01, Fig. 4d). The second significant SNP (chr5.S_198197360, *P*-value = 1.80E-07, R^2^ = 9.8%) locates in the third intron of GRMZM2G044851 (Fig. 4b), encoding the nitrate transporter 1.5. The average root hair length for allele with A was greatly longer than allele with C (*P* ≤ 0.01, Fig. 4e). The third significant SNP (chr6.S_ 75630277, *P*-value = 3.97E-07, *R*^2^ = 9.7%), which is the one with other 7 SNPs within the same LD region, locates at the promoter region of GRMZM5G825276 (Fig. 4c), encoding a GDSL-like lipase. The average root hair length for allele with A was greatly longer than allele with G (*P* ≤ 0.01, Fig. 4f). It is noted that the haplotype analysis for the other eight genes was depicted in Fig. S2.

## Discussion

Root hairs enlarge the root surface area and thereby play vital roles in plant absorbing water and nutrient, as well as coping with biotic and abiotic stress. In recent years, extensive mutation-based studies have been carried out to dissect the genetic regulatory network determining root hair development, but we lack fundamental knowledge about whether and how root hair is controlled in a plant natural variation population. In this work, we interpreted the natural variation and the associated genetic architecture of root hair length in a maize association panel, and a set of putative candidate genes controlling root hair development were revealed by performing GWAS analysis.

### Phenotypic variability and coordination of root hair length with other processes of plant development and growth

Maize was domesticated from its wild ancestor, teosinte (*Zea mays ssp. parviglumis*) about 8000–9000 years ago. Nowadays, modern maize displays a large geographic distribution from tropical to temperate climate. Therefore, population structure is associated with significant differences in maize morphology [51]. In this study, we observed that root hair exhibits wide length variation with normal distribution. Interestingly, tropical lines displayed shorter root hairs relative to temperate lines, implying that the adaption of maize from tropical to temperate regions was probably accompanied by the character of lessening root hair growth.

In our investigation, the root hair at the length dimension was negatively rather than positively correlated with several morphological and metabolic traits. This is somewhat counter-intuitive. However, it is known that the maize plant is a monocot and has a fibrous root system consisting of massive adventitious and lateral roots, the anatomy of which is markedly different from dicotyledonous model Arabidopsis [52–54]. In this context, it is possible that the other aspects of root system may enable maize to have adequate larger surface area to support plant growth under normal soil condition. Meanwhile, it is worthy to be mention that to facilitate high throughput phenotyping, the root hair from the emerging primary roots after 3-days post germination was assessed in this study, thereby whether the growth rate will alter distinctly in different inbred lines is uncertain at this point.

### Putative genes involved in root hair morphogenesis

We identified a total of 11 candidate genes associated with root hair elongation, and the homologs of three genes have been reported to influence root hair development in *Arabidopsis*. GRMZM2G100288 encodes a receptor-like protein kinase, and its closest homologs in *Arabidopsis* is *FERONIA* (*FER*), which is well known as a key hub of cell signaling networks mediating various hormone, stress, and immune responses [55–65]. Root hair initiation and elongation require functional FER, and its loss-of-function caused severe root hair defects [66–68]. *GRMZM2G064644* encodes a protein containing a CRIB motif required for its specific interaction with GTP-bound Rop1 (plant-specific Rho GTPase), promoting root hair development [69–71]. *GRMZM2G403003* encoding a TON1 recruiting motif (TRM) proteins is able to target TON1 to cortical microtubules [72]. It has been reported that the disruption of transverse cortical microtubules is essential for root hair initiation [73, 74].

Nucleotide binding site leucine-rich repeat (NBS–LRR) comprises a large class of disease resistance proteins that play a widespread role in plant protection against pathogens [75, 76]. Surprisingly, out of 11 candidate genes associated with root hair elongation, 3 encode NBS-LRR protein. Although disease resistance is the only function demonstrated for NBS-LRR proteins so far, their functions in other areas of plant biology cannot be excluded [77]. For instance, Arabidopsis *BNT1* encodes an atypical TIR-NBS-LRR protein and works as a regulator of the hormonal response to stress. The disruption of BNT1 could result in dramatic alteration in root hair distribution [78].

The function and morphology of root hairs in relation to the acquisition of water and nutrients have been well studied previously [79]. Root hair length could be stimulated and elongated under different nutrient deficiencies, such as phosphorus, potassium, magnesium, iron, or manganese [80–84]. *GRMZM2G000471* and *GRMZM2G044851* encode an aquaporin and a nitrate transporter, respectively. The loss-of-function of *HvEXPB7* and *OsWOX11* severely suppressed root hairs under drought conditions [85, 86]. AtNPF7.3/NRT1.5 has been reported to alter lateral root architecture under potassium deprivation [87].

Root hair requires robust activity of various metabolism pathways to support its polar elongation [24, 88–90]. *GRMZM2G147446* encodes a UDP-rhamnose/UDP-galactose transporter situated in the Golgi lumen where UDP-galactose is used for synthesis of noncellulosic polysaccharides and glycoproteins [91–94]. *GRMZM5G825276* encodes a GDSL-like lipase involved in lipid biosynthesis [95, 96]. Overall, although the involvement of these candidate genes in root hair growth and development is biologically conceivable, their biological importance waits further functional validation. Meanwhile, once functionally proved, how the natural polymorphisms in these genes regulating root hair growth are the other intriguing questions worthy of future studies. Moreover, given that the importance of root hair in plant growth, especially under water or nutrient limitation condition, understanding and manipulating the molecular regulatory network of root hair development would prospectively benefit and facilitate the crop breeding program.

## Conclusions

Prior to this study, genetic architecture and genes controlling natural variation in maize root hair development remains unclear. We elucidated a broadly natural variation in the root hair length in a maize association population. Several genetic loci putatively regulating the natural variation in root hair were revealed by performing GWAS, shedding light on novel knowledge about the genetic basis of root hair development in maize. Given that the importance of root hair in plant nutrient acquisition and water uptake, candidate genes identified in this study provide a list of research targets for future functional characterization to understand how root hair growth is naturally regulate, and benefit the breeding program to improve maize varieties with proper root hair morphology assisted by molecular breeding and engineering.

## Materials and methods

### Association mapping panel and genotyping

Association tests were conducted in an association panel consisting of 281 inbred lines, which are publicly available at http://maizego.org/. The maize lines in this association panel were clustered into three subpopulations, including 22 SS lines, 30 NSS lines and 112 TST lines, and the remaining 117 lines fall into a mixed subpopulation. Detailed information about the origins of these lines was described in previous studies [42, 43]. The 1.25 million high-quality SNPs (MAF > 5%) and the estimated population structure and kinship were assessed using the method previously described [97, 98].

### Phenotypic data collection and statistical analysis

Maize seeds were surface-sterilized in a 10% NaClO solution for 5 min, rinsed several times with distilled water, and germinated in moist germination paper rolls (Anchor Paper) at 28 °C under constant darkness as previously described [25]. The root hair length of primary roots from 3-day-old plants were captured using stereo microscope. Quantification of the root hair length was measured from captured images using ImageJ. Given that the limited number of inbred lines could be measured at the same time, we cultured 15 inbred lines in each batch, and selected 5 representative plants at the same developmental stage to measure the root hair length. To rule out the variation, the B73 line was used as control of each batch. Eventually, the ratio of root hair length between each individual inbred line and B73 were collected and inputted as phenotypic data.

### Genome-wide association mapping and phenotypic variance contribution of significant loci

A GWAS on root hair length was performed using Tassel 5.2 under both GLM and MLM. Considering the non-independence of SNPs caused by strong LD, it is usually too strict for significant association detection when the threshold is derived from the total number of markers. Thus, the effective number of independent markers for the multiple adjustment were used to obtain the *P* value thresholds [99, 100]. The 165,248 markers in approximate linkage equilibrium with each other were found by PLINK (window size 50, step size 50, *r*^*2*^ ≥ 0.2) [101]. Then, we used the uniform Bonferroni-corrected thresholds at α = 1 for MLM and α = 0.05 for GLM as the significance cutoffs as reported in the previous studies [37, 44, 102]. Finally, the suggestive *P* value was computed by 1/n and 0.05/n (*n* = 165,248), and we obtained the suggestive threshold 6.05 × 10^− 6^ for MLM and 3.03 × 10^− 7^ for GLM, respectively.

To estimate the phenotypic variance explained by each significant SNP, we used ANOVA to construct linear models of *Y* = *αX* + *βP* + *ε* (1) and *Y* = *βP* + *ε* (2). In this model, *Y* is the phenotype, *X* is the SNP genotype, *P* is the matrix of three subpopulations (NSS, SS and TST), *α* is the SNP effect, *β* is the subpopulation effects, and ε is random effects. Thus, the R^2^ of each significant SNP after adjusting for the population structure effects were reported as previously described [37].

### Pathway analysis

The pathway analysis was performed in https://maizegdb.org/past. The resulting SNP-trait association data and effects data generated by TASSEL were implemented in the pathway analysis. During the process of loading data, the LD data is filtered to drop rows where the loci are not the same, and then unneeded columns from the TASSEL output are dropped [103]. Only pathways with five or more mapped genes were considered in the analysis. Significance of the enrichment score was determined by permutation analysis (1000 random permutations of the effect values).

### Prediction of candidate genes

To search the candidate genes underlying associated SNPs, we selected the most significant/leading SNPs within the same LD block (*R*^2^ < 0.2) to represent the locus, and candidate genes were nominated by the leading SNP positioned. The physical locations of the SNPs were recorded according to the B73 RefGen_v2 (www.maizesequence.org). The corresponding genes were annotated based on the literatures describing the function of their homologs in other species or the information retrieved from conserved domain database (CDD).

## Supplementary Information

**Additional files 1: Figure S1**. GWAS showing significant *P*-values associated with root hair length based on the general linear model (GLM). (a) Manhattan plots of GLM for root hair length. The horizontal dashed line represents the significance threshold -log10(*P*) = 6.5. (b) QQ plot of GLM showing the ratio of the observed and the expected *P*-values for root hair length. The solid diagonal lines represent agreement between observed and expected probability distributions assuming null SNP–trait association.

**Additional files 2: 0Figure S2**. The allele effects of significant SNPs located around representative genes for root hair length. (a–d; i-l) Regional plots showing association mapping results for SNPs located around *GRMZM2G100288* (a), *GRMZM2G147446* (b), *GRMZM2G078013* (c), *GRMZM2G000471* (d), *GRMZM2G064644* (i), *AC193598.3_FG002* (j), *GRMZM2G180244* (k), *GRMZM2G091579* (l). (e–h; m-p) Allele effects of the most significant SNPs for root hair traits. (e) *GRMZM2G100288*, (f) *GRMZM2G147446*, (g) *GRMZM2G078013*, (h) *GRMZM2G000471*, (m) *GRMZM2G064644*, (n) *AC193598.3_FG002*, (o) *GRMZM2G180244*, (p) *GRMZM2G091579*. Each dot represents an SNP. The horizontal dashed black line represents the significant threshold –log10(*P*) = 5.2. *Significant at *P* ≤ 0.05; **significant at *P* ≤ 0.01.

**Additional files 3: Table S1**. Root hair length ratio (Inbreds/B73) of the 281 inbred lines from different subpopulations.

**Additional files 4: Supplemental Table 2**. Pathways associated with increased root hair length at statistical significance *P* < 0.05.

**Additional files 5: Supplemental Table 3**. Pathways associated with decreased hair length at statistical significance *P* < 0.05.

## Data Availability

All supporting data can be found within the manuscript and its additional files.

## Abbreviations

CW: Cob weight
DTA: Days to anthesis
DTH: Days to heading
DTS: Days to silking
ED: Ear diameter
EH: Ear height
EL: Ear length
ELL: Ear leaf length
ELW: Ear leaf width
GLM: General linear model
GW: Cob grain weight
GWAS: Genome-wide association study
K matrix: Kinship relationship
KNPR: Kernel number per row
KW: Kernel width
LD: Linkage disequilibrium
LNAE: Leaf number above ear
MAF: Minor allele of frequency
MLM: Mixed linear model
PH: Plant height
Q matrix: Population structure
R^2^: Percentage of phenotypic variation explained by the identified SNPs
SNPs: Single nucleotide polymorphisms
TBN: Tassel branch number
TMAL: Tassel maximum axis length

## References

[1] Petersen LN, Ingle RA, Knight MR, Denby KJ (2009). OXI1 protein kinase is required for plant immunity against *Pseudomonas syringae* in *Arabidopsis*. J Exp Bot.

[2] Grierson C, Nielsen E, Ketelaarc T, Schiefelbein J (2014). Root hairs. Arabidopsis Book.

[3] Gilroy S, Jones DL (2000). Through form to function: root hair development and nutrient uptake. Trends Plant Sci.

[4] Lee RDW, Cho HT. Auxin, the organizer of the hormonal/environmental signals for root hair growth. Front Plant Sci. 2013;4. 10.3389/fpls.2013.00448. 10.3389/fpls.2013.00448 PMC3824147 24273547

[5] Nestler J, Keyes SD, Wissuwa M (2016). Root hair formation in rice (*Oryza sativa* L.) differs between root types and is altered in artificial growth conditions. J Exp Bot.

[6] Salazar-Henao JE, Vélez-Bermúdez IC, Schmidt W (2016). The regulation and plasticity of root hair patterning and morphogenesis. Development..

[7] Cui SK, Suzaki T, Tominaga-Wada R, Yoshida S (2018). Regulation and functional diversification of root hairs. Semin Cell Dev Biol.

[8] Berger F, Hung CY, Dolan L, Schiefelbein J (1998). Control of cell division in the root epidermis of *Arabidopsis thaliana*. Dev Biol.

[9] Balcerowicz D, Schoenaers S, Vissenberg K (2015). Cell fate determination and the switch from diffuse growth to planar polarity in *Arabidopsis* root epidermal cells. Front Plant Sci.

[10] Bruex A, Kainkaryam RM, Wieckowski Y, Kang YH, Bernhardt C, Xia Y, Zheng X, Wang JY, Lee MM, Benfey P, Woolf PJ, Schiefelbein J (2012). A gene regulatory network for root epidermis cell differentiation in Arabidopsis. PLoS Genet.

[11] Gu F, Nielsen E (2013). Targeting and regulation of cell wall synthesis during tip growth in plants. J Integr Plant Biol.

[12] Marzec M, Melzer M, Szarejko I (2015). Root hair development in the grasses: what we already know and what we still need to know. Plant Physiol.

[13] Kim CM, Han CD, Dolan L (2017). RSL class I genes positively regulate root hair development in *Oryza sativa*. New Phytol.

[14] Moon S, Cho LH, Kim YJ, Gho YS, Jeong HY, Hong WJ, Lee C, Park H, Jwa NS, Dangol S, Chen Y, Park H, Cho HS, An G, Jung KH (2019). RSL class II transcription factors guide the nuclear localization of RHL1 to regulate root hair development. Plant Physiol.

[15] Ma N, Wang Y, Qiu S, Kang Z, Che S, Wang G, et al. Overexpression of *OsEXPA8*, a root-specific gene, improves rice growth and root system architecture by facilitating cell extension. PloS One. 2013;8(10):e75997. 10.1371/journal.pone.0075997 PMC3790854 24124527

[16] Yu Z, Kang B, He X, Lv S, Bai Y, Ding W, Chen M, Cho H, Wu P (2011). Root hair-specific expansins modulate root hairelongation in rice. Plant J.

[17] Kim CM, Park SH, Il Je B, Park SH, Park SJ, Piao HL, Eun MY, Dolan L, Han CD (2007). *OsCSLD1*, a cellulose synthase-like D1 gene, is required for root hair morphogenesis in rice. Plant Physiol.

[18] Huang J, Kim CM, Xuan YH, Liu J, Kim TH, Kim BK, Han CD (2013). Formin homology 1 (OsFH1) regulates root-hair elongation in rice (*Oryza sativa*). Planta..

[19] Huang J, Kim CM, Xuan YH, Park SJ, Piao HL, Je BI, Liu J, Kim TH, Kim BK, Han CD (2013). *OsSNDP1*, a Sec14-nodulin domain-containing protein, plays a critical role in root hair elongation in rice. Plant Mol Biol.

[20] Guo M, Ruan W, Li C, Huang F, Zeng M, Liu Y, Yu Y, Ding X, Wu Y, Wu Z, Mao C, Yi K, Wu P, Mo X (2015). Integrative comparison of the role of the PHOSPHATE RESPONSE1 subfamily in phosphate signaling and homeostasis in Rice. Plant Physiol.

[21] Wen TJ, Hochholdinger F, Sauer M, Bruce W, Schnable PS (2005). The *roothairless1* gene of maize encodes a homolog of *sec3*, which is involved in polar exocytosis. Plant Physiol.

[22] Hochholdinger F, Wen TJ, Zimmermann R, Chimot-Marolle P, da Costa e Silva O, Bruce W, Lamkey KR, Wienand U, Schnable PS (2008). The maize (*Zea mays* L.) *roothairless3* gene encodes a putative GPI-anchored, monocot-specific, COBRA-like protein that significantly affects grain yield. Plant J.

[23] Nestler J, Liu S, Wen TJ, Paschold A, Marcon C, Tang HM, Li D, Li L, Meeley RB, Sakai H, Bruce W, Schnable PS, Hochholdinger F (2014). *Roothairless5*, which functions in maize (*Zea mays* L.) root hair initiation and elongation encodes a monocot-specific NADPH oxidase. Plant J.

[24] Li L, Hey S, Liu S, Liu Q, McNinch C, Hu HC, et al. Characterization of maize *roothairless6* which encodes a D-type cellulose synthase and controls the switch from bulge formation to tip growth. Sci Rep. 2016;6:34395. 10.1038/srep34395 PMC5052636 27708345

[25] Wang C, Qi C, Luo J, Liu L, He Y, Chen L (2019). Characterization of *LRL5* as a key regulator of root hair growth in maize. Plant J.

[26] Zhang X, Mi Y, Mao H, Liu S, Chen L, Qin F (2020). Genetic variation in *ZmTIP1* contributes to root hair elongation and drought tolerance in maize. Plant Biotechnol J.

[27] Yu J, Buckler ES (2006). Genetic association mapping and genome organization of maize. Curr Opin Biotechnol.

[28] Atwell S, Huang YS, Vilhjálmsson BJ, Willems G, Horton M, Li Y, Meng D, Platt A, Tarone AM, Hu TT, Jiang R, Muliyati NW, Zhang X, Amer MA, Baxter I, Brachi B, Chory J, Dean C, Debieu M, de Meaux J, Ecker JR, Faure N, Kniskern JM, Jones JDG, Michael T, Nemri A, Roux F, Salt DE, Tang C, Todesco M, Traw MB, Weigel D, Marjoram P, Borevitz JO, Bergelson J, Nordborg M (2010). Genome-wide association study of 107 phenotypes in *Arabidopsis thaliana* inbred lines. Nature..

[29] Huang X, Zhao Y, Wei X, Li C, Wang A, Zhao Q, Li W, Guo Y, Deng L, Zhu C, Fan D, Lu Y, Weng Q, Liu K, Zhou T, Jing Y, Si L, Dong G, Huang T, Lu T, Feng Q, Qian Q, Li J, Han B (2011). Genome-wide association study of flowering time and grain yield traits in a worldwide collection of rice germplasm. Nat Genet.

[30] Riedelsheimer C, Lisec J, Czedik-Eysenberg A, Sulpice R, Flis A, Grieder C, Altmann T, Stitt M, Willmitzer L, Melchinger AE (2012). Genome-wide association mapping of leaf metabolic profiles for dissecting complex traits in maize. Proc Natl Acad Sci U S A.

[31] Gore MA, Chia JM, Elshire RJ, Sun Q, Ersoz ES, Hurwitz BL, Peiffer JA, McMullen MD, Grills GS, Ross-Ibarra J (2009). A first-generation haplotype map of maize. Science..

[32] Yan J, Shah T, Warburton ML, Buckler ES, McMullen MD, Crouch J. Genetic characterization and linkage disequilibrium estimation of a global maize collection using SNP markers. PLoS ONE. 2009;4(12):e8451. 10.1371/journal.pone.0008451 PMC2795174 20041112

[33] Kump KL, Bradbury PJ, Wisser RJ, Buckler ES, Belcher AR, Oropeza-Rosas MA, Zwonitzer JC, Kresovich S, McMullen MD, Ware D (2011). Genome-wide association study of quantitative resistance to southern leaf blight in the maize nested association mapping population. Nat Genet.

[34] Guo Z, Tucker DM, Wang D, Basten CJ, Ersoz E, Briggs WH, Lu J, Li M, Gay G (2013). Accuracy of across-environment genome-wide prediction in maize nested association mapping populations. G3 (Bethesda).

[35] Leiboff S, Li X, Hu HC, Todt N, Yang J, Li X, et al. Genetic control of morphometric diversity in the maize shoot apical meristem. Nat Commun. 2015;6:8974. 10.1038/ncomms9974 PMC4673881 26584889

[36] Cui Z, Luo J, Qi C, Ruan Y, Li J, Zhang A, et al. Genome-wide association study (GWAS) reveals the genetic architecture of four husk traits in maize. BMC Genomics. 2016;17(1):946. 10.1186/s12864-016-3229-6 PMC5117540 27871222

[37] Wang X, Wang H, Liu S, Ferjani A, Li J, Yan J, Yang X, Qin F (2016). Genetic variation in ZmVPP1 contributes to drought tolerance in maize seedlings. Nat Genet.

[38] Li X, Zhou Z, Ding J, Wu Y, Zhou B, Wang R, et al. Combined linkage and association mapping reveals QTL and candidate genes for plant and ear height in maize. Front Plant Sci. 2016;7. 10.3389/fpls.2016.00833. 10.3389/fpls.2016.00833 PMC4908132 27379126

[39] Zhang X, Guan Z, Wang L, Fu J, Zhang Y, Li Z, Ma L, Liu P, Zhang Y, Liu M, Li P, Zou C, He Y, Lin H, Yuan G, Gao S, Pan G, Shen Y (2019). Combined GWAS and QTL analysis for dissecting the genetic architecture of kernel test weight in maize. Mol Gen Genomics.

[40] Jia T, Wang L, Li J, Ma J, Cao Y, Lübberstedt T, Li H (2019). Integrating a genome-wide association study with transcriptomic analysis to detect genes controlling grain drying rate in maize (*Zea may*, L.). Theor Appl Genet.

[41] Li W, Yu Y, Wang L, Luo Y, Peng Y, Xu Y, Liu X, Wu S, Jian L, Xu J, Xiao Y, Yan J (2021). The genetic architecture of the dynamic changes in grain moisture in maize. Plant Biotechnol J.

[42] Yang X, Gao S, Xu S, Zhang Z, Prasanna BM, Li L, Li J, Yan J (2010). Characterization of a global germplasm collection and its potential utilization for analysis of complex quantitative traits in maize. Mol Breeding.

[43] Li H, Peng Z, Yang X, Wang W, Fu J, Wang J, Han Y, Chai Y, Guo T, Yang N, Liu J, Warburton ML, Cheng Y, Hao X, Zhang P, Zhao J, Liu Y, Wang G, Li J, Yan J (2012). Genome-wide association study dissects the genetic architecture of oil biosynthesis in maize kernels. Nat Genet.

[44] Yang N, Lu Y, Yang X, Huang J, Zhou Y, Ali F, et al. Genome wide association studies using a new nonparametric model reveal the genetic architecture of 17 agronomic traits in an enlarged maize association panel. PLoS Genet. 2014;10(9):e1004573. 10.1371/journal.pgen.1004573 PMC4161304 25211220

[45] Deng M, Li D, Luo J, Xiao Y, Liu H, Pan Q, Zhang X, Jin M, Zhao M, Yan J (2017). The genetic architecture of amino acids dissection by association and linkage analysis in maize. Plant Biotechnol J.

[46] Tang JD, Perkins A, Williams WP, Warburton ML (2015). Using genome-wide associations to identify metabolic pathways involved in maize aflatoxin accumulation resistance. BMC Genomics.

[47] Warburton ML, Womack ED, Tang JD, Thrash A, Smith JS, Xu WW, et al. Genome-wide association and metabolic pathway analysis of corn earworm resistance in maize. Plant Genome. 2018;11(1):170069. 10.3835/plantgenome2017.08.0069 PMC12962561 29505629

[48] Li H, Thrash A, Tang JD, He L, Yan J, Warburton ML (2019). Leveraging GWAS data to identify metabolic pathways and networks involved in maize lipid biosynthesis. Plant J.

[49] Yu J, Pressoir G, Briggs WH, Vroh Bi I, Yamasaki M, Doebley JF, McMullen MD, Gaut BS, Nielsen DM, Holland JB, Kresovich S, Buckler ES (2005). A unified mixed-model method for association mapping that accounts for multiple levels of relatedness. Nat Genet.

[50] Zhang Z, Ersoz E, Lai CQ, Todhunter RJ, Tiwari HK, Gore MA, Bradbury PJ, Yu J, Arnett DK, Ordovas JM, Buckler ES (2010). Mixed linear model approach adapted for genome-wide association studies. Nat Genet.

[51] Castelletti S, Coupel-Ledru A, Granato I, Palaffre C, Cabrera-Bosquet L, Tonelli C, et al. Maize adaptation across temperate climates was obtained via expression of two florigen genes. PloS Genet. 2020;16(7):e1008882. 10.1371/journal.pgen.1008882 PMC7386623 32673315

[52] Hochholdinger F (2004). Genetic dissection of root formation in maize (*Zea mays*) reveals root-type specific developmental programmes. An Bot.

[53] Hochholdinger F, Park WJ, Sauer M, Woll K (2004). From weeds to crops: genetic analysis of root development in cereals. Trends Plant Sci.

[54] Petricka JJ, Winter CM, Benfey PN (2012). Control of *Arabidopsis* root development. Annu Rev Plant Biol.

[55] Guo H, Li L, Ye H, Yu X, Algreen A, Yin Y (2009). Three related receptor-like kinases are required for optimal cell elongation in *Arabidopsis thaliana*. Proc Natl Acad Sci U S A.

[56] Deslauriers SD, Larsen PB (2010). FERONIA is a key modulator of brassinosteroid and ethylene responsiveness in *Arabidopsis* hypocotyls. Mol Plant.

[57] Kessler SA, Shimosato-Asano H, Keinath NF, Wuest SE, Ingram G, Panstruga R, Grossniklaus U (2010). Conserved molecular components for pollen tube reception and ungal invasion. Science..

[58] Stegmann M, Monaghan J, Smakowska-Luzan E, Rovenich H, Lehner A, Holton N, et al. The receptor kinase FER is a RALF-regulated scaffold controlling plant immune signaling. Science. 2017;355(6322):287–9. 10.1126/science.aal2541 28104890

[59] Gommers CMM, Keuskamp DH, Buti S, van Veen H, Koevoets IT, Reinen E, Voesenek LACJ, Pierik R (2017). Molecular profiles of contrasting shade response strategies in wild plants: differential control of immunity and shoot elongation. Plant Cell.

[60] Barbez E, Dünser K, Gaidora A, Lendl T, Busch W (2017). Auxin steers root cell expansion via apoplastic pH regulation in *Arabidopsis thaliana*. Proc Natl Acad Sci U S A.

[61] Feng W, Kita D, Peaucelle A, Cartwright HN, Doan V, Duan Q, Liu M-C, Maman J, Steinhorst L, Schmitz-Thom I, Yvon R, Kudla J, Wu HM, Cheung AY, Dinneny JR (2018). The FERONIA receptor kinase maintains cell-wall integrity during salt stress through Ca^2+^ signaling. Curr Biol.

[62] Zhao C, Zayed O, Yu Z, Jiang W, Zhu P, Hsu CC, Zhang L, Tao WA, Lozano-Durán R, Zhu J-K (2018). Leucine-rich repeat extensin proteins regulate plant salt tolerance in *Arabidopsis*. Proc Natl Acad Sci U S A.

[63] Dong Q, Zhang Z, Liu Y, Tao LZ, Liu H (2018). FERONIA regulates auxin-mediated lateral root development and primary root gravitropism. FEBS Lett.

[64] Guo H, Nolan TM, Song G, Liu S, Xie Z, Chen J, Schnable PS, Walley JW, Yin Y (2018). FERONIA receptor kinase contributes to plant immunity by suppressing Jasmonic acid signaling in *Arabidopsis thaliana*. Curr Biol.

[65] Wang L, Yang T, Wang B, Lin Q, Zhu S, Li C, et al. RALF1-FERONIA complex affects splicing dynamics to modulate stress responses and growth in plants. Sci Adv. 2020;6(21):eaaz1622. 10.1126/sciadv.aaz1622 PMC7314565 32671204

[66] Duan Q, Kita D, Li C, Cheung AY, Wu HM (2010). FERONIA receptor-like kinase regulates RHO GTPase signaling of root hair development. Proc Natl Acad Sci U S A.

[67] Zhu S, Estévez JM, Liao H, Zhu Y, Yang T, Li C, Wang Y, Li L, Liu X, Pacheco JM, Guo H, Yu F (2020). The RALF1–FERONIA complex phosphorylates eIF4E1 to promote protein synthesis and polar root hair growth. Mol Plant.

[68] Zhu S, Martínez Pacheco J, Estevez JM, Yu F (2020). Autocrine regulation of root hair size by the RALF-FERONIA-RSL4 signaling pathway. New Phytol.

[69] Molendijk AJ, Bischoff F, Rajendrakumar CSV, Friml J, Braun M, Gilroy S, Palme K (2001). *Arabidopsis thaliana* Rop GTPases are localized to tips of root hairs and control polar growth. EMBO J.

[70] Vernoud V, Horton AC, Yang Z, Nielsen E (2003). Analysis of the small GTPase gene superfamily of Arabidopsis. Plant Physiol.

[71] Denninger P, Reichelt A, Schmidt VAF, Mehlhorn DG, Asseck LY, Stanley CE, Keinath NF, Evers J-F, Grefen C, Grossmann G (2019). Distinct RopGEFs successively drive polarization and outgrowth of root hairs. Curr Biol.

[72] Drevensek S, Goussot M, Duroc Y, Christodoulidou A, Steyaert S, Schaefer E, Duvernois E, Grandjean O, Vantard M, Bouchez D, Pastuglia M (2012). The *Arabidopsis* TRM1-TON1 interaction reveals a recruitment network common to plant cortical microtubule arrays and eukaryotic centrosomes. Plant Cell.

[73] Takahashi H, Hirota K, Kawahara A, Hayakawa E, Inoue Y (2003). Randomization of cortical microtubules in root epidermal cells induces root hair initiation in lettuce (*Lactuca sativa* L.) seedlings. Plant Cell Physiol..

[74] Takahashi H, Kawahara A, Inoue Y (2003). Ethylene promotes the induction by auxin of the cortical microtubule randomization required for low-pH-induced root hair initiation in lettuce (*Lactuca sativa* L.) seedlings. Plant Cell Physiol.

[75] Zhao B, Lin X, Poland J, Trick H, Leach J, Hulbert S (2005). A maize resistance gene functions against bacterial streak disease in rice. Proc Natl Acad Sci U S A.

[76] Zhang C, Chen H, Cai T, Deng Y, Zhuang R, Zhang N, Zeng Y, Zheng Y, Tang R, Pan R, Zhuang W (2017). Overexpression of a novel peanut NBS-LRR gene *AhRRS5* enhances disease resistance to *Ralstonia solanacearum* in tobacco. Plant Biotechnol J.

[77] McHale L, Tan X, Koehl P, Michelmore RW. Plant NBS-LRR proteins: adaptable guards. Genome Biol. 2006;7(4):212. 10.1186/gb-2006-7-4-212 PMC1557992 16677430

[78] Sarazin V, Duclercq J, Mendou B, Aubanelle L, Nicolas V, Aono M, Pilard S, Guerineau F, Sangwan-Norreel B, Sangwan RS (2015). *Arabidopsis BNT1*, an atypical *TIR–NBS–LRR* gene, acting as a regulator of the hormonal response to stress. Plant Sci.

[79] Tanaka N, Kato M, Tomioka R, Kurata R, Fukao Y, Aoyama T, Maeshima M (2014). Characteristics of a root hair-less line of *Arabidopsis thaliana* under physiological stresses. J Exp Bot.

[80] Bates TR, Lynch JP (1996). Stimulation of root hair elongation in *Arabidopsis thaliana* by low phosphorus availability. Plant Cell Environ.

[81] Schikora A, Schmidt W (2001). Iron stress-induced changes in root epidermal cell fate are regulated independently from physiological responses to low iron availability. Plant Physiol.

[82] Yang TJW, Perry PJ, Ciani S, Pandian S, Schmidt W (2008). Manganese deficiency alters the patterning and development of root hairs in *Arabidopsis*. J Exp Bot.

[83] Jung JY, Shin R, Schachtman DP (2009). Ethylene mediates response and tolerance to potassium deprivation in *Arabidopsis*. Plant Cell.

[84] Niu Y, Chai R, Liu L, Jin G, Liu M, Tang C, Zhang Y (2014). Magnesium availability regulates the development of root hairs in *Arabidopsis thaliana* (L.) Heynh. Plant Cell Environ.

[85] He X, Zeng J, Cao F, Ahmed IM, Zhang G, Vincze E, Wu F (2015). *HvEXPB7*, a novel β-expansin gene revealed by the root hair transcriptome of Tibetan wild barley, improves root hair growth under drought stress. J Exp Bot.

[86] Cheng S, Zhou D, Zhao Y. *WUSCHEL*-related homeobox gene *WOX11* increases rice drought resistance by controlling root hair formation and root system development. Plant Signal Behav. 2016;11(2):e1130198. 10.1080/15592324.2015.1130198 PMC4883865 26689769

[87] Zheng Y, Drechsler N, Rausch C, Kunze R. The Arabidopsis nitrate transporter NPF7.3/NRT1.5 is involved in lateral root development under potassium deprivation. Plant Signal Behav. 2016;11(5):e1176819. 10.1080/15592324.2016.1176819 PMC4973756 27089248

[88] Diet A, Link B, Seifert GJ, Schellenberg B, Wagner U, Pauly M, Reiter WD, Ringli C (2006). The *Arabidopsis* root hair cell wall formation mutant *lrx1* is suppressed by mutations in the *RHM1* gene encoding a UDP-L-rhamnose synthase. Plant Cell.

[89] Wang X (2005). Regulatory functions of phospholipase D and phosphatidic acid in plant growth, development, and stress responses. Plant Physiol.

[90] Kf de Campos M, Schaaf G (2017). The regulation of cell polarity by lipid transfer proteins of the SEC14 family. Curr Opin Plant Biol.

[91] Reyes F, Orellana A (2008). Golgi transporters: opening the gate to cell wall polysaccharide biosynthesis. Curr Opin Plant Biol.

[92] Rautengarten C, Ebert B, Moreno I, Temple H, Herter T, Link B, Donas-Cofre D, Moreno A, Saez-Aguayo S, Blanco F, Mortimer JC, Schultink A, Reiter WD, Dupree P, Pauly M, Heazlewood JL, Scheller HV, Orellana A (2014). The Golgi localized bifunctional UDP-rhamnose/UDP-galactose transporter family of *Arabidopsis*. Proc Natl Acad Sci U S A.

[93] Orellana A, Moraga C, Araya M, Moreno A (2016). Overview of nucleotide sugar transporter gene family functions across multiple species. J Mol Biol.

[94] Temple H, Saez-Aguayo S, Reyes FC, Orellana A (2016). The inside and outside: topological issues in plant cell wall biosynthesis and the roles of nucleotide sugar transporters. Glycobiology..

[95] Akoh CC, Lee GC, Liaw YC, Huang TH, Shaw JF (2004). GDSL family of serine esterases/lipases. Prog Lipid Res.

[96] Clauß K, Baumert A, Nimtz M, Milkowski C, Strack D (2008). Role of a GDSL lipase-like protein as sinapine esterase in Brassicaceae. Plant J.

[97] Liu H, Luo X, Niu L, Xiao Y, Chen L, Liu J, Wang X, Jin M, Li W, Zhang Q, Yan J (2017). Distant eQTLs and non-coding sequences play critical roles in regulating gene expression and quantitative trait variation in maize. Mol Plant.

[98] Gui S, Yang L, Li J, Luo J, Xu X, Yuan J, et al. ZEAMAP, a comprehensive database adapted to the maize Multi-Omics era. iScience. 2020;23(6):101241. 10.1016/j.isci.2020.101241 PMC7306594 32629608

[99] Li MX, Yeung JMY, Cherny SS, Sham PC (2011). Evaluating the effective numbers of independent tests and significant *p*-value thresholds in commercial genotyping arrays and public imputation reference datasets. Hum Genet.

[100] Yang W, Guo Z, Huang C, Duan L, Chen G, Jiang N, et al. Combining high-throughput phenotyping and genome-wide association studies to reveal natural genetic variation in rice. Nat Commun. 2014;5:5087. 10.1038/ncomms6087 PMC4214417 25295980

[101] Purcell S, Neale B, Todd-Brown K, Thomas L, Ferreira MAR, Bender D, Maller J, Sklar P, de Bakker PIW, Daly MJ, Sham PC (2007). PLINK: a tool set for whole-genome association and population-based linkage analyses. Am J Human Genet.

[102] Mao H, Wang H, Liu S, Li Z, Yang X, Yan J, et al. A transposable element in a *NAC* gene is associated with drought tolerance in maize seedlings. Nat Commun. 2015;6:8326. 10.1038/ncomms9326 PMC4595727 26387805

[103] Thrash A, Tang JD, DeOrnellis M, Peterson DG, Warburton ML. PAST: The Pathway Association Studies Tool to infer biological meaning from GWAS datasets. Plants (Basel). 2020;9(1):58. 10.3390/plants9010058 PMC7020396 31906457

