# Supplementary material for: Genome-wide association study reveals the genetic architecture of root hair length in maize
Source: BMC Genomics. 2021 Sep 14;22:664. doi: 10.1186/s12864-021-07961-z (PMC8442424; doi:10.1186/s12864-021-07961-z)
Supplement: Supplementary file 1 — Additional files 1: Figure S1. GWAS showing significant P-values associated with root hair length based on the general linear model (GLM). (a) Manhattan plots of GLM for root hair length. The horizontal dashed line represents the significance threshold -log10(P) = 6.5. (b) QQ plot of GLM showing the ratio of the observed and the expected P-values for root hair length. The solid diagonal lines represent agreement between observed and expected probability distributions assuming null SNP–trait association. [file 12864_2021_7961_MOESM1_ESM.pdf]

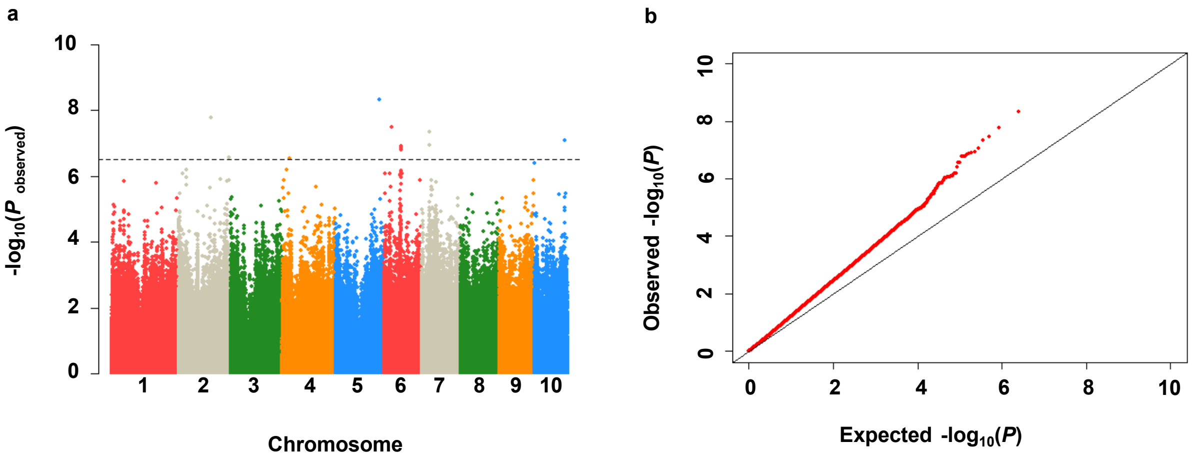

Figure S1. GWAS showing significant *P*-values associated with root hair length based on the general linear model (GLM).

(a) Manhattan plots of GLM for root hair length. The horizontal dashed line represents the significance threshold -log10(*P*) = 6.5.

(b) QQ plot of GLM showing the ratio of the observed and the expected *P*-values for root hair length. The solid diagonal lines represent agreement between observed and expected probability distributions assuming null SNP−trait association.
