# Supplementary material for: Genome-wide association study reveals the genetic architecture of root hair length in maize
Source: BMC Genomics. 2021 Sep 14;22:664. doi: 10.1186/s12864-021-07961-z (PMC8442424; doi:10.1186/s12864-021-07961-z)
Supplement: Supplementary file 2 — Additional files 2: 0Figure S2. The allele effects of significant SNPs located around representative genes for root hair length. (a–d; i-l) Regional plots showing association mapping results for SNPs located around GRMZM2G100288 (a), GRMZM2G147446 (b), GRMZM2G078013 (c), GRMZM2G000471 (d), GRMZM2G064644 (i), AC193598.3_FG002 (j), GRMZM2G180244 (k), GRMZM2G091579 (l). (e–h; m-p) Allele effects of the most significant SNPs for root hair traits. (e) GRMZM2G100288, (f) GRMZM2G147446, (g) GRMZM2G078013, (h) GRMZM2G000471, (m) GRMZM2G064644, (n) AC193598.3_FG002, (o) GRMZM2G180244, (p) GRMZM2G091579. Each dot represents an SNP. The horizontal dashed black line represents the significant threshold –log10(P) = 5.2. *Significant at P ≤ 0.05; **significant at P ≤ 0.01. [file 12864_2021_7961_MOESM2_ESM.pdf]

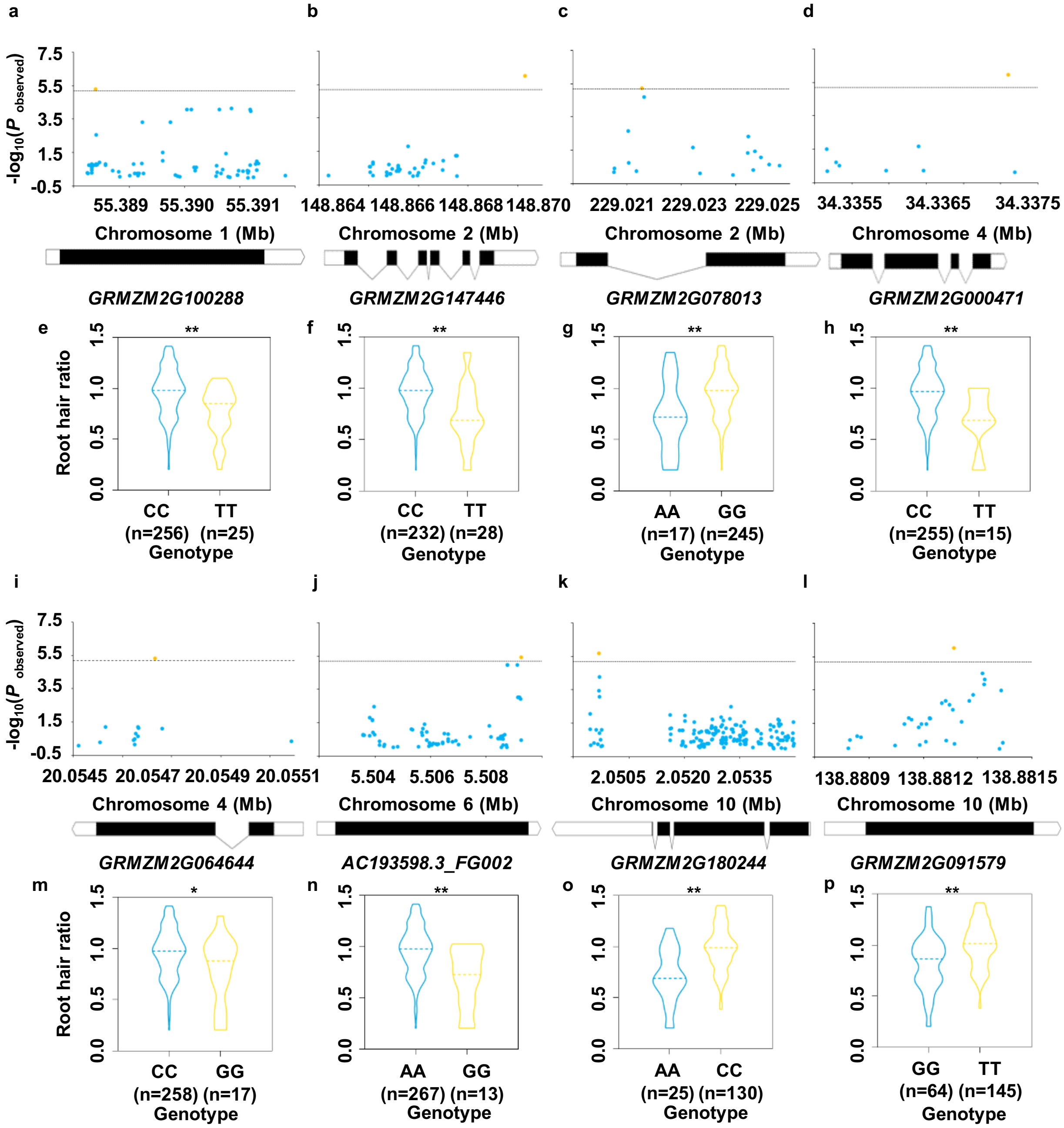

Figure S2. The allele effects of significant SNPs located around representative genes for root hair length.
(a–d; i-l) Regional plots showing association mapping results for SNPs located *around GRMZM2G100288* (a), *GRMZM2G147446* (b), *GRMZM2G078013* (c), *GRMZM2G000471* (d), *GRMZM2G064644* (i), *AC193598.3_FG002* (j), *GRMZM2G180244* (k), *GRMZM2G091579* (l).
(e–h; m-p) Allele effects of the most significant SNPs for root hair traits. (e) *GRMZM2G100288*, (f) *GRMZM2G147446*, (g) *GRMZM2G078013*, (h) *GRMZM2G000471*, (m) *GRMZM2G064644*, (n) *AC193598.3_FG002*, (o) *GRMZM2G180244*, (p) *GRMZM2G091579*. Each dot represents an SNP. The horizontal dashed black line represents the significant threshold –log10(P) = 5.2. *Significant at $P \leq 0.05$; **significant at $P \leq 0.01$.
